# Putting explainable AI in context: institutional explanations for medical AI

**DOI:** 10.1007/s10676-022-09649-8

**Published:** 2022-05-06

**Authors:** Mark Theunissen, Jacob Browning

**Affiliations:** 1 New York University, New York, USA; 2 Department of Values, Technology and Innovation, School of Technology, Policy and Management, Delft University of Technology, Delft, The Netherlands

**Keywords:** AI and health, Explainable AI, Ethical design, Epistemic risk

## Abstract

There is a current debate about if, and in what sense, machine learning systems used in the medical context need to be explainable. Those arguing in favor contend these systems require post hoc explanations for each individual decision to increase trust and ensure accurate diagnoses. Those arguing against suggest the high accuracy and reliability of the systems is sufficient for providing epistemic justified beliefs without the need for explaining each individual decision. But, as we show, both solutions have limitations—and it is unclear either address the epistemic worries of the medical professionals using these systems. We argue these systems do require an explanation, but an *institutional* explanation. These types of explanations provide the reasons why the medical professional should rely on the system in practice—that is, they focus on trying to address the epistemic concerns of those using the system in specific contexts and specific occasions. But ensuring that these institutional explanations are fit for purpose means ensuring the institutions designing and deploying these systems are transparent about the assumptions baked into the system. This requires coordination with experts and end-users concerning how it will function in the field, the metrics used to evaluate its accuracy, and the procedures for auditing the system to prevent biases and failures from going unaddressed. We contend this broader explanation is necessary for either post hoc explanations or accuracy scores to be epistemically meaningful to the medical professional, making it possible for them to rely on these systems as effective and useful tools in their practices.

## Introduction

There have always been technologies that are understood by few but trusted by many, and there is a long history of technologies which functioned before anyone understood the mechanics behind them. This is especially the case in the history of medicine, an ancient field which only in recent decades has developed comprehensive theories of physical health and evidence-based medicine. Given this history of the field, it might seem surprising there is a vocal demand for explanation in relation to one particular medical technology: artificial intelligence systems. But there are at least three important reasons machine learning (ML) systems pose their own problems in the medical context. First, whereas other technologies often work as tools to supplement medical care, ML often is providing diagnostic decisions—functioning as a “second opinion.”^1^ Second, the systems often use counterintuitive and inexplicable methods for diagnosis, leveraging massive amounts of data to detect minute, highly abstract relationships between variables which no doctor would think to correlate. Third, many ML systems are designed to be statistical pattern-recognizers, functioning at the level of correlations, with no sense of which patterns are relevant and which are spurious. This suggests ML systems pose risks other technologies do not pose. The hope has been that explainable AI (XAI) might be able to alleviate these problems by providing useful explanations of what the system is doing when deciding one way or the other.

While engineers have been providing explanations of varying sorts for these systems, the central problem is designing an *appropriate* explanation. Most have aimed to provide a *post* *hoc explanation* for why a specific decision resulted from specific inputs. This is a technical engineering solution for providing insight into why particular inputs were evaluated one way rather than another. But whether these technological solutions are accurate or useful is an open question, and some researchers, such as London (2019), have argued instead that we should ignore explanations and instead focus on creating the most accurate algorithms possible.

In this paper, we argue that discussions of XAI have been misplaced, focusing on *technological* solutions—ensuring accuracy or providing post hoc explanations—that often do little to resolve the epistemic issues these systems raise. Accuracy scores, while obviously important, cannot on their own make a system reliable (Dotan, 2020). And as Zednick (2021) has noted, many of the post hoc explanations are most useful to the engineers designing the system (e.g., preventing gross error or overfitting) while proving less useful for end-users who need to integrate these systems into their practices. A different kind of explanation is needed for minimizing the epistemic risks connected with relying on black-box systems, one that makes clear why the system is reliable, appropriate for its task, and carefully calibrated to its situation. This second kind of explanation, what we call an *institutional explanation,* is essential for justifying the usage of these machines by medical professionals. These are more pragmatic explanations, communicating information about the machine that aims to alleviate the epistemic concerns of the medical professionals relying on it (Nyrup & Robinson, 2022). Calling these explanations institutional is highlighting that they are addressing the design decisions that went into the making of the system just as much as they are the technical specifications of the system: why should we trust the company and engineers designing it, what efforts have they been made to avoid bias, and how have they worked alongside end-users who will use these systems in their medical practice? We show that this kind of explanation—focused especially on the human decisions involved in and a part of the design and deployment of these systems—is essential both for interpreting accuracy scores and ensuring post hoc explanations are meaningful to end-users. For either of those to be epistemically useful for medical practitioners, they ultimately depend on an institutional explanation.

In the next section we discuss some of the issues surrounding medical AI and known problems around providing adequate post hoc explanations. Specifically, we highlight that these explanations do not necessarily justify a user’s trust in the system. In section two, we focus on the view that accuracy is sufficient for treating ML systems as reliable and some of the limitations of that view. In section three, we introduce the idea of an institutional explanation by highlighting the limits of the post hoc explanation or accuracy debate. In section four, we highlight how institutional explanations, in order to epistemically justify usage of the systems for the medical professional must also ensure the institution deploying them is responsible for their accuracy over time. In section five, we contend that the institutional explanations need to address the non-epistemic values embodied in the system’s design, and how these should shape the way medical professionals use these systems to prevent paternalism or overtreatment.

## Thinking about explainability

The explanatory challenge facing the ML engineer is that the use of big data increasingly leads to extraordinarily complex systems. ML systems solve problems in what Burrell (2016) calls “methodologically opaque” ways: “deep” multilayer networks with thousands of parameters discovering incredibly nuanced statistical patterns in a vast trove of data. The result is multiple layers of opacity with little insight into how the results of any layer are achieved or which specific patterns are being detected. While it is possible to provide different degrees of methodological transparency (Creel, 2020), in many cases this transparency does little to increase our understanding of why the system decides one way or the other (Sullivan, 2019).

This appears to suggest explainability is impossible to achieve and we should either abandon methodologically opaque algorithms (Holzinger et al., 2017) or abandon the quest for explanation by accepting the proven high accuracy of these algorithms as sufficient justification for using them despite a lack of the desired post hoc explanation (London, 2019). But before taking any stance, it is important to note that all explanation is contextual: a go grandmaster interested in why AlphaGo made a specific move is not interested in the technological explanation; they want to know what strategy the machine is using—that is, an intentional level description of the behavior (Zerilli et al., 2019). For many ML systems being deployed in the sciences, the appropriate explanations are those which are reliable and valid, the first requiring post hoc explanation, the later convergence with other systems and models (Durán, 2021). For medical professionals, however, the desire for an explanation often rests on a mistrust of these algorithms, especially in cases where they regard the system's diagnoses as competing with rather than complimenting their own work (Genin & Grote, 2021). There is thus a justifiable worry about relying on the algorithm since it might well lead to misdiagnosis (Gaube et al., 2021). As such, the explanation needed is one which provides grounds for *relying on* the system’s output—for trusting the system as a useful tool in the medical context. The contextual nature of explanation is important because, as Zednik (2021) has effectively shown, different post hoc explanations are not just warranted for different agents, but also not all agents will be able to understand or use just any explanation. This latter point is important, because a misfit between agent and post hoc explanation can lead to problematic results: an accurate result with a confusing explanation might lead a user to wrongly distrust a system, whereas an inaccurate result with a clear explanation can lead to unwarranted trust. Even in less problematic cases, it is also possible the post hoc explanation is simply unhelpful to the agent—providing an explanation about how something works at the wrong level of detail, for example.

The issue of choosing the right explanation appropriate to the agent within the context of application is important to foreground because many kinds of XAI are geared towards *engineers—*or, at least, people already familiar with the technology. Lipton (2018) provides an excellent overview of the different approaches for addressing a systems opacity—transparency, interpretability, and explainability—as well as a critical analysis of their different strengths and weaknesses. These are typically post hoc explanations which provide some analysis of the most salient features which predict the result. These technical explanations can be essential for stakeholders, such as the experts establishing specifications for the machine or engineers trying to optimize it. Thus many post hoc explanations are designed for helping engineers grasp whether the system is doing what is supposed to: ensuring the system is tracking causal factors, generalizing in appropriate ways, or making fair and unbiased decisions. Different post hoc explanations can be essential for engineers in detecting gross error and determining interventions to correct flaws in the model.

The kinds of explanations designed for engineers, however, are typically less useful to the end-users. Many of these approaches turn on technical details of the ML system and the target domain; even the most intuitive of such approaches, such as confidence scores or the weighted assessment of the most salient features in the input, usually depend on some ability to evaluate statistics and some understanding of the background domain. For the lay person, there often needs to be an explanation for the explanation, so to speak. The takeaway is that none of these approaches provide all the information necessary; there is no all-purpose explanation which satisfies all agents in all contexts.

This points to a deeper problem facing the use of ML systems: their methodological opacity leads to an *epistemic* opacity which post hoc explanations do not necessarily help resolve. If a technical explanation is confusing or deceptively simple, it is plausible that it reduces epistemic transparency in some cases and in others increases transparency without warrant. This is especially important when one understands how post hoc explanations are made. ML “explanations” or “interpretations” tend to involve creating a second, different ML system to interpret or explain the outputs of the first system. But the result of this process means the end-user is required to rely on one ML system to explain another ML system, with potential worries about the reliability of both. As Durán & Jongsma note, “The fundamental problem with transparency is, to our mind, that it is itself based on opaque processes. Indeed, transparency displaces the question of opacity of *A* to the question of opacity of *P*” (2021, p. 331). Creating transparent, interpretable systems does not necessarily improve the epistemological opacity of the system relative to those who stand in need of a certain sort of account about an ML’s working.

But the lack of a post hoc explanation is not necessarily a barrier to epistemic transparency. As an example, consider an ML system detecting melanoma from images (Esteva et al., 2017). As Sullivan (2019) highlights, the medical professional knows how the system works on one level: visual appearances are the primary method of detecting melanoma and the ML system is a visual recognition system trained on skin samples, and thus there is a clear, comprehensible link what the machine is detecting and how it is detecting it (2019, pp. 21–23). And background information—that it was trained on primarily light-skinned individuals and thus likely it less reliable on darker skin-tones—is part of how the system is presented. This information provides an understanding of not just how the machine has been trained and why it is reliable, but also lays out in which contexts and for which patients its use is not recommended. In short, an appropriate and useful explanation need not involve post hoc explanations at all, since explaining other features of the machine—how it works, what contexts it works in, how it was trained—may be sufficient for justifying its usage. It is also, in large part, the same kind of explanation a doctor would make to defend their own reliability: they were trained by looking at pictures while under the guidance of a supervisor, their training and background provide sufficient guidance for deciding cases, they are most comfortable detecting certain skin conditions on certain types of skin, and they cannot rule out mistakes but are much more reliable than not (Engel, 2008; Polanyi, 1958).

The takeaway is that it is not necessarily the case that post hoc explanations increase epistemic grounds for relying on the machine in the first place. And it is also not necessarily the case that a lack of post hoc explanation makes a machine untrustworthy.

## The insufficiency of accuracy

The last section showed some of the problems surrounding post hoc explanations. One response has been to reject the need for post hoc explanations altogether. London (2019) argues that the requirement of accuracy of ML systems renders the need for explainability less pressing or even moot. Critics of the accuracy approach respond that, since ML can only detect correlations without causation, the lack of interpretability makes them a dangerous tool that could dissipate patient’s trust in doctors and the medical field (Grote & Berens, 2020). And Rudin (2019) argues moreover that, in principle, any inexplicable ML system can be replaced by one that is interpretable. London counters, however, that there is a tradeoff in the other direction as well: interpretable models often require far longer to train. He writes,

As counterintuitive and unappealing as it may be, the opacity, independence from an explicit domain model, and lack of causal insight associated with some of the most powerful machine learning approaches are not radically different from routine aspects of medical decision-making. Our causal knowledge is often fragmentary, and uncertainty is the rule rather than the exception. In such cases, careful empirical validation of an intervention’s practical merits is the most important task**.** When the demand for explanations of how interventions work is elevated above careful, empirical validation, patients suffer, resources are wasted, and progress is delayed. (London, 2019, 18)

^2021^).

But whether an ML system is accurate is not a straightforward question. As Dotan (2020) notes, predictive accuracy is not sufficient for justifying using one ML system over another, since lower accuracy on a training set is compatible with much higher accuracy on non-training data. Thus, ensuring a system is accurate in the appropriate sense involves weighing other factors, both epistemic and non-epistemic. Durán and Formanek (2018) have proposed their “computational reliabilism” framework to provide a more thorough framework for comparing different models to ensure a system is more accurately tracking real features relevant to the user. The computational reliabilism framework has more recently been extended to the medical field (Durán & Jongsma, 2021). This framework should be regarded as essential for developing useful and reliable systems—effectively best practices for those developing ML systems. It ensures systems are not just thoroughly verified on training data (and thus accurate in a narrow sense), but also validated on non-training samples and tested against competitors and predecessors to ensure it receives comparable results when appropriate (i.e., is not overfitting or detecting spurious correlations). Computational reliabilism also consults experts at all stages of the design of the system, ensuring the epistemic and non-epistemic concerns of those who will use the system are effectively integrated.

We take computational reliabilism as being essential for ensuring the accuracy of a system is robust and properly formed with expert input. However, this approach focuses primarily on how engineer’s design and test the technology in the lab, with less recognition of how it needs to be integrated into the medical practice. This concern with the technological design understates the challenge of making the machine a useful tool for the medical professional in context, which requires a more *robust* validation—one that makes clear it is working appropriately in the field. The example of Google’s ML system to detect diabetic retinopathy from images of patients’ eyes is salutary: the system had been thoroughly trained on high-quality, well lit, perfectly centered images. The resulting system had a roughly human 90% accuracy rate. But, in the real world, where many images are off center, poorly lit, or slightly blurry, the machine was far less effective or even incapable of reading them. The result was a highly accurate system that rarely worked and ended up being a burden to the nurses tasked with using it (Heaven, 2020). The problem here is not technological, but an institutional gap between those designing ML systems and those using them.

The upshot is that technologically-focused approaches to ML systems are unlikely, on their own, to properly address the problems. In the next section we argue what is needed is a human-focused attempt to render the *institution* of designing and deploying algorithms more reliable and, in turn, comprehensible for those using these systems.

## Institutional explanations

The problem with the debate between accuracy or post hoc explanation approaches is that both base the burden of justifying these systems on simple, easy-to-use, technology-centered metrics. This fails to address the gap between lab and real-world that has proven challenging to close in the medical field (Heaven, 2021). While physicians in general are optimistic AI can be integrated successfully into their work, trusting individual systems and their results has proven more difficult (Sarwar et al., 2019). Accuracy scores or heatmaps are simply not capable of bearing the weight of turning AI into reliable tools for the medical domain, since they leave unaddressed why the medical professionals should trust the *humans* who designed the systems.

As Helen Nissenbaum notes, if patients trust the medical professionals to perform a delicate surgery, it is not because they have been given an adequate consent form that explains what precautions will be taken or what risks are involved. Rather, the consent form is taken as sufficient precisely because they trust the *institution* of medicine, a hard-won trust involving “the long years of study and apprenticeship that physicians undergo, the state and board certifications, peer oversight, professional codes, and above all, the system’s interest (whatever the source) in our well-being” (2011, p. 36). The consent form functions because we already trust the institution; if we did not trust it, the consent form would not increase our trust. In the same way, accuracy scores and post hoc explanations will increase epistemic transparency only if we already have good grounds for trusting the institution designing and deploying the system.

The intent behind providing an institutional explanation is an attempt to make explicit some of the processes, safeguards, and commitments made by the humans designing and deploying these systems. The hope is that these explanations can reduce the epistemic opacity of these systems and provide grounds for the medical professionals relying on these systems. The goal is for the ML designers to provide a fuller account, making explicit to the medical professional why the system is reliable in vivo, fleshing out both the epistemic and non-epistemic values which oriented its development and the overall strategies to ensure it works in the contexts the professionals are facing. This aims to provide evidence for the competence and trustworthiness of those designing the system, which thus can ground the medical professional treating the system as reliable.

Concretely, an institutional explanation provides the medical professional with the background epistemic and non-epistemic values built into a system and how they shape both how the system works and why it is reliable, but also how medical professionals should use it. This functions as an explanation for what the institution aimed to accomplish with the system, how medical professionals and the medical context shaped the design, and what efforts are made to render it accurate—both at present and over time. Explaining these features aims to directly address the epistemic opacity by addressing what efforts the humans involved in the design and deployment of the system have taken to ensure the system is an appropriate fit, to ensure the designers of the system took into consideration the kinds of concerns the medical professional is concerned with.

To clarify how an institutional explanation can help, it is useful to consider a well-discussed example in the literature—one that has been used to support both post hoc explanations and accuracy-only approaches. Caruana et al. (2015) discusses a neural network that predicted asthmatics have a lower probability of death from pneumonia than the population as a whole. This struck the medical professionals as inaccurate since asthmatics are, in fact, most endangered from lung conditions such as pneumonia. Thus, the system was broadly ignored by the professionals, which Caruna takes as evidence that a post hoc explanation was necessary. However, as London responds to Caruana’s et al. account, the neural network is accurate; asthmatics *are* more likely to recover, not because pneumonia is less dangerous for them, but instead because they receive more immediate and extensive medical care throughout. London writes, “the system is actuarially correct—patients with asthma *who receive aggressive medical intervention* have a lower probability of death than some non-asthmatic patients who likely receive less aggressive medical care” (2019, p. 19). The problem is thus that the system is accurate but is perceived as inaccurate because the doctors have different starting assumptions about what a predictive algorithm should provide. The medical professionals assume the machine is predicting outcomes assuming a shared baseline of care, not differential amounts of care relative to the background of the patient.

Resolving this situation cannot rely simply on the machine being accurate if medical professionals do not understand it. But, *pace* Caruna, it also would not be solved simply with a post hoc explanation. In the case of the most typical post hoc explanations—for example, the most predictive features responsible for a certain decision (Creel, 2020, p. 583)—the result would not decrease epistemic opacity: it would rightly rate the prior condition, asthma, as the most predictive factor in the decision that they are unlikely to die from pneumonia. There is no single factor which might correspond to “aggressive medical intervention,” since this is not a single thing but instead many different factors which would individually not be very predictive but only when taken in the aggregate. In short, it is unlikely an interpretable system would have helped the befuddled doctors any more than an accuracy report from the engineers.

Caruana’s et al. example helps clarify how an institutional explanation can address these issues. The system becomes more reliable when agent, machine and context are properly calibrated—in this case, by ensuring medical professionals recognize the baseline for predictions is the normal amount of care indicated for a specific patient. This requires the engineers sufficiently understanding what their system is doing, something that cannot be accomplished without close collaboration with experts, both in the general design of the algorithm but also by testing it on site. This onsite collaboration is essential since the kinds of knowledge possessed by engineers and experts is each relative to their own field, meaning there needs to be a close collaboration for both groups to understand the machine—for the engineers to grasp what kinds of patterns the ML system is picking out, and for the medical professionals to grasp how the outputs should be applied to concrete cases.

Although this is a simple point, it is one that has been easily missed because the general approach has been to treat medical AI systems as *adversaries* to the medical professionals (Grote & Berens, 2020). As a result, the focus has been on automating the skillful judgments of professionals, rather than designing and deploying ML systems that address the epistemic concerns of those who need to work alongside these systems. Institutional explanations are designed to assuage the professionals’ doubts about the reliability of the machine stemming from concerns that the institutions designing medical AI cannot be trusted to understand the technical and contextual issues involved in the use of these systems. The explanation will need to explain how the ML system was made to be appropriate for medical practice, which requires establishing the success the system showed in making the medical professionals more reliable in the real-world—processes which are, as yet, not standard or even expected for deploying these systems (Staff, 2021). Since the problems arising out of real-world interactions are likely to only be discovered as the system is used in more cases and more contexts, this requires the design of an algorithm involve extensive onsite collaboration between engineers and medical professionals.

The upshot is that institutional explanations can provide the grounds for rendering accuracy scores useful for the medical professionals by indicating what they mean in terms of the professionals’ own practice. In the next section, we highlight the importance of *re-*calibration—regular tests and audits of the system—in ensuring medical professionals can trust the system is being monitored over time.

## The variability of risk

Shifting the focus in the XAI debates away from technological explanations and towards institutional explanations helps highlight other problems facing using ML systems in medical contexts. While validating a system is essential for ensuring the system works on real-world data in a general sense, the medical context is not static. As more patients are examined, there is a constant possibility that the algorithm will encounter situations which do not resemble its training data—not just because of biased or insufficient training data, but also because the world changes.

A very recent example of this was made visible in the recent COVID-19 pandemic when not just the virus itself continued to mutate but also, in its explosive growth, it began to infest a far more diverse selection of people. This posed very different epistemic risks for medical professionals making difficult diagnoses about who would require intensive care treatment and who would be capable of riding out the miserable symptoms with less intensive care or at home. Early in the pandemic, many hospitals opted for Epic’s ML predictive algorithm to help assess which patients would likely experience rapid deterioration despite the fact the technology was not independently tested (Khetpal & Shah, 2021).

The ethics of this decision are vexing, but the justification for deploying the system was that it was released in a low-information environment where many hospitals encountered no patients with COVID-19 before they suddenly encountered many at once. This led to the uncomfortable choice between choosing a machine which had not been independently tested or relying on hearsay and studies not yet peer-reviewed on medRxiv and bioRxiv—or, like the hydroxychloroquine paper in the *Lancet,* insufficiently reviewed. In a situation where there are no good options, the epistemic risk of embracing the algorithm early in the pandemic is arguably as high as the alternatives. However, eight months into the pandemic, when medical professionals became more familiar with the virus, after more peer-reviewed studies came out, when best practices developed, and when both the virus itself and the profile of those infected changed drastically, it was no longer adequate in terms of minimizing epistemic risks to continue relying on the machine without independent testing. The epistemic risk of continuing to rely on the machine became higher as medical professionals’ understanding of COVID, its diagnosis, and its treatment all changed.^2^

This example highlights the limits of technology-focused approaches. The initial accuracy score provided plausible grounds for deploying and relying on the system, but this score was established early in the pandemic based on a limited data sample. As the pandemic grew—and began to affect a broader swathe of people demographically—it becomes increasingly necessary to audit the system to test whether it was still accurate. The same institutional explanation which provides reasons to trust the system at the beginning ceases to be compelling eight months later, since the algorithm remained static while the situation had changed radically. The risk of relying on the machine changed over time and audits were essential for ensuring it remained reliable as more information is gathered. This shows that the validation of a system is something that cannot be completed all at once but is an open-ended ongoing process (re)establishing that the machine is (still) reliable.

Similar issues about the variability of risks over time also occur when dealing with the exposure of technology to a more diverse population. This is not just a problem for algorithms, of course. In the case of the pandemic, this problem was especially visible with the rapid deployment of COVID vaccines and in the use of pulse oximeters. Two recent COVID-19 vaccines, by Astra-Zeneca and Johnson and Johnson respectively, proved remarkably effective at preventing infection and serious illness from COVID-19. However, both revealed an increased incidence of blood clots in patients, with a higher incidence in women (Garcia de Jesús, 2021). Similarly, the pulse oximeter proved an essential tool for measuring oxygen saturation levels in patients and determining on that basis who is at higher risk of serious illness. Pulse oximeters are devices applied to a patient’s finger which passes a light through the finger with the output read on the other side. The variability of the light which passes through indicates the amount of oxygen in the blood. The technology was tested out in a predominantly white community (Bickler, 2005), and a study published in late 2020 (Sjoding et al., 2020) highlighted significant variability depending on skin color with the measurement of the oxygen saturation levels: darker skinned individuals were typically misrepresented as having higher levels of oxygen, and thus in fact being at higher risk, than light skinned individuals with the same oxygenation level as measured by arterial blood gas. In short, pulse oximeters often overestimated oxygen saturation levels in people with darker skin.

These examples provide a key insight: ensuring these systems are reliable is challenging and will not be accomplished in the laboratory but will likely not appear until they are deployed in context. While the accuracy of the ML system will matter in this context, what is more important is the human choices behind dealing with an uncertain situation. But an explanation at this level is also one that should make clear that the situation is uncertain and in what ways; in the COVID case, the uncertainty stems from limited datasets taken on a single variant within a short timeframe. For the institutional explanation to actually limit epistemic risk, it also requires providing some guidelines for when the system will be audited (and possibly replaced) on a more representative dataset. The need for regular auditing—for detecting potential problems and ensuring they are properly logged and addressed—must be not only built into the usage of these systems but also explained to practitioners as an essential feature of the cooperation of professional and machine. This is necessary for discovering biases in the system and picking out what proxies for bias (Johnson, 2020), as in the oximeter case, are responsible for the problems. Ensuring that communities with a history of neglect and abuse by the medical community trust these technologies requires the institutional explanations to explicitly address what measures were taken to ensure the system was fit for use—or, if measures were not taken (because of limited sample size of a rare disease, for example), why the medical professional should exercise more skepticism in certain cases. Regular auditing is essential for ensuring the system works well, but an institutional explanation expressing the uncertainty in the system design and an account for how the audits are addressing them is essential for these systems to explain why these systems are worth relying on for those using them.

The upshot is that the institutional explanation should not only establish why the system is taken to be reliable at a given moment, but also how the institution is ensuring the machine will remain reliable over time—either by updating or replacing it.

## Non-epistemic values and risks

The last sections focused on how the institutional explanation makes explicit how these systems address the epistemic risks involved in using them, firstly by accounting for their calibration to the concerns of medical experts and, secondly by making explicit the uncertainty involved in the design of the system and what efforts are made to lessen it over time. This final section shifts the focus to how institutional explanation is also intended to address the non-epistemic risks involved in designing and using these systems.

Integrating any technology into regular usage in medical practice is not merely an epistemic matter; ethically engineering and deploying these systems must also involve integrating and collaborating with the numerous stakeholders affected by the technology—and doing so requires attention to non-epistemic values. These involve integrating multiple stakeholders, designing systems around their usefulness to practitioners, and ensuring the system will respect the autonomy of users and provide just outputs. As Biddle (2016, 2020; see also Biddle & Kukla, 2017) shows, the choice of what metrics and thresholds are chosen to diagnose a patient poses risks which require value judgments which cannot be decided solely by medical professionals without threatening paternalism and overtreatment. Approaches for designing these algorithms must integrate conceptual work done by philosophers, engineers, and social theorists with various forms of empirical research from data science to ethnography, even direct collaboration with primary users and patients to have the design process and result adequately support their values. This is because numerous non-epistemic values—not just safety but also values like accessibility or intuitiveness—play essential roles in ensuring technology proves a help rather than a hindrance to those providing medical care. These broader factors are increasingly understood to be essential for designing ethical, integrated engineered products, as seen in the “value sensitive design” approach for AI (Friedman & Hendry, 2019; Umbrello & Van Der Poel, 2021).

However, this highlights the dimension that will become the most challenging for filling in institutional explanations. Specifically, designers will need to work far more closely with both medical professionals and ethicists for ensuring the systems produced address the dangers of this kind of technology for those being subjected to it (Grote & Berens, 2020). In the medical setting, ensuring the machine is reliable addresses the beneficence and nonmaleficence values for medical care, but it does not address concerns about patient autonomy or whether they are being dealt with justly (Beauchamp & Childress, 2019). These problems can only partially be addressed by further evaluation and regular auditing; an entirely accurate and understood machine that does not help patients make informed decisions is incompatible with their autonomy. The medical professional needs not only epistemic justification for relying on the machine, but also the tools to address patients’ concerns about the machine. This is especially important since many ML systems, such as those providing triage decisions for overstaffed hospitals, are unlikely to be discussed with patients who are having their fate determined by them. Even if the algorithms are reliable, there are still ethical concerns about paternalism and patient autonomy in these systems becoming invisible parts of medical practice.

This points to why institutional explanations are not only necessary, but also why they need to be pragmatic, with emphasis on the need for them to be communicated in a way the medical professional can relate to patients (Nyrup & Robinson, 2022). It needs to be the case that the medical professional understands how the machine is impacting the patient and what steps are necessary for properly informing them about its usage—but also integrating into an ethics-centered medical practice. An anecdotal example of the issue provided to one of the authors concerns an ML system responsible for predicting the chances of cancer and its responsiveness to chemotherapy. After a grueling series of chemotherapy treatments resulting in the removal of the cancer, a friend was told the system predicted that a further round would significantly lower the likelihood of recurrence. While the medical professionals relied on and deferred to the machine, they could provide no further information about it—how it was trained, which cancers it was most reliable on, what factors it was making the evaluation based on, and when it was last evaluated. However, the patient found the professionals *very* pushy—paternalistically arguing that the machine’s decision was sufficient for justifying another round. This highlights that they were *not* provided with an appropriate explanation for how the machine worked, and thus could not successfully communicate about the machine or permitting them from providing helpful information about how the machine works (Sand et al., 2022). The professional’s reliance on the machine also clearly resulted in a failure to respect the autonomy of the patient, since she was clearly weighing non-epistemic concerns—just how miserable chemo is and whether it would be an unacceptable loss of quality of life.

The upshot is that many of the concerns about XAI are better understood as challenges facing the justification of this new kind of tool, and merely technological solutions will not be sufficient to justify them. What is necessary is a more integrated practice of developing and deploying these systems, one which results in a pragmatically useful explanation for why these new tools should be relied on by medical professionals.

## Conclusion

We have argued that approaches focused on post hoc explanations or accuracy scores for ML systems are overly complex technologies like ML are overly narrow in the medical context. This is because neither is likely to ensure the algorithm is relied on by the medical professional, much less those affected by the technology. Instead, the appropriate approach is an institutional explanation, where the accuracy of the system is not only shown to be reliable from the engineer’s perspective but also reliable from the medical professional’s perspective. The latter demands human–machine calibration, which should provide an explanation of when, how, and why the algorithm is to be used. But this approach cannot establish that the technology functions properly all at once; the explanation is meant to be limited, acknowledging its uncertainty and making explicit how often it will be audited and reassessed in order to ensure the uncertainty in the system is, both for the continuing reliability of the system for the medical professional but also for justifying its usage to patients—many of whom have legitimate reasons to distrust the medical field and medical technology.

An important takeaway is that it is misguided to think there is one sort of ideal explanation or accuracy score for these complex systems independent of the social context of their deployment. Even if a post hoc explanation is called for, a context-sensitive and appropriate algorithm will ultimately need to be one that addresses the concerns of practitioners, and that likely will not be known until the system is evaluated in context. Providing an institutional explanation for when, how, and why the system is reliable ultimately grounds the post hoc explanations. In short, it will be because the medical professionals regard the system as institutionally reliable that the post hoc explanations are useful, not vice-versa.

## Footnotes

1 Benjamens et al. (2020) provide a helpful chart on the various ML systems currently in use in the United States, as well as the appropriate regulations governing these devices. Many, but far from all, have no diagnostic role but instead streamline or simplify aspects of medical practice. These, we take it, are largely not interesting from the perspective of the philosophy and ethics of AI.

2 This is also when the algorithm did receive independent testing (Singh et al., 2020).
